# Rebleeding rate after interventional therapy directed by capsule endoscopy in patients with obscure gastrointestinal bleeding

**DOI:** 10.1186/1471-230X-8-12

**Published:** 2008-04-23

**Authors:** Hiroki Endo, Nobuyuki Matsuhashi, Masahiko Inamori, Keiko Akimoto, Tomohiko Ohya, Tatsuro Yanagawa, Masako Asayama, Kantaro Hisatomi, Takuma Teratani, Koji Fujita, Masato Yoneda, Atsushi Nakajima

**Affiliations:** 1 Division of Gastroenterology, Yokohama City University School of Medicine, Yokohama, Japan; 2 Department of Gastroenterology, Kanto Medical Center NTT EC, Tokyo, Japan

## Abstract

**Background:**

The precise role of capsule endoscopy in the diagnostic algorithm of obscure gastrointestinal bleeding has yet to be determined. Despite the higher diagnostic yield of capsule endoscopy, the actual impact on clinical outcome remains poorly defined. The aim of this study was to evaluate the follow-up results of patients with obscure gastrointestinal bleeding to determine which management strategies after capsule endoscopy reduced rebleeding.

**Methods:**

All patients in whom the cause of obscure gastrointestinal bleeding was investigated between May 2004 and March 2007 were studied retrospectively. We evaluated the clinical outcome of patients with obscure gastrointestinal bleeding after capsule endoscopy using the rebleeding rate as the primary outcome.

**Results:**

Seventy-seven patients with obscure gastrointestinal bleeding underwent capsule endoscopy. Capsule endoscopy identified clinically significant findings that were thought to be the sources of obscure gastrointestinal bleeding in 58.4% of the patients. The overall rebleeding rate was 36.4%. The rebleeding rate was significantly higher among patients with insignificant findings than among those with significant findings (*p *= 0.036). Among the patients in whom capsule endoscopy produced significant findings, the rebleeding rate of the patients who underwent therapeutic interventions was significantly lower than that in those who did not undergo intervention (9.5% vs 40.0%, *p *= 0.046).

**Conclusion:**

Follow-up and further aggressive interventions are necessary for patients with obscure gastrointestinal bleeding and significant capsule endoscopy findings to reduce the chance of rebleeding.

## Background

Obscure gastrointestinal bleeding (OGIB) is a common problem encountered by gastroenterologists. OGIB is defined as bleeding of unknown origin that persists or recurs after a negative initial evaluation [1], and accounts for approximately 5% of all gastrointestinal (GI) bleeding [2]. Many cases of OGIB originate in the small bowel, where the source of bleeding can be difficult to detect. Capsule endoscopy (CE) has revolutionized the evaluation of OGIB [3,4]. CE is now a well-accepted non-invasive modality for evaluating diseases of the small intestine [4-7]. CE has been shown to be superior to push enteroscopy [8,9], small bowel follow-through [10], and computed tomography [11-13] for detecting bleeding lesions in the small intestine. Most published studies on CE have focused on the diagnostic yield, although a few have focused on clinical outcome – including the rebleeding rate of patients after CE examination [14,15]. One study suggested that the influence of CE on clinical outcome was rather low despite the high diagnostic yield [16]. Data on whether CE affects patient outcome are sparse. CE can reveal various findings of the small bowel, but no specific guidelines exist for the management of lesions detected by CE. Whether follow-up and further aggressive interventions are necessary for patients after CE remains controversial. The aim of this study was to evaluate the follow-up results of patients with OGIB who underwent CE to determine which management strategies after CE reduced rebleeding.

## Methods

All patients who were referred to the Kanto Medical Center NTT EC to undergo CE for the investigation of OGIB between May 2004 and March 2007 were evaluated retrospectively. All of the patients had visible passage of blood or positive fecal occult blood testing with a drop in hemoglobin. Most of the patients had undergone a total colonoscopy, gastroscopy, and small bowel follow-through examination prior to undergoing CE. Written informed consent to the CE procedure was obtained from all the patients. The exclusion criteria were a suspected small bowel obstruction, stricture, swallowing disorders, pacemaker implantation, and pregnancy.

All videos were reviewed using the PillCam SB capsule endoscopy system (Given Imaging Ltd., Israel). CE was performed after a 12-hour fasting period. Fluid and light meals were allowed 2 and 4 hours after capsule swallowing. The patients were free to leave the hospital, with instructions to return within the 8-hour study period to have the data recorder removed. The recorded digital information was downloaded from the recorder into the computer and the images were analyzed using the proprietary RAPID software. All CE images were reviewed by two experienced gastroenterologists. The CE findings were classified as either clinically significant or clinically insignificant findings. Examples of clinically significant findings included ulcers, angiodysplasias, tumors, bleeding without identifiable lesion, or Crohn's disease. Examples of clinically insignificant findings include erosions, small ulcers, red spots, small polyps, or negative findings. The definitions for clinically significant or clinically insignificant findings have been standardized at our institution. Significant findings were defined as the presence of CE findings that might account for the clinical bleeding.

Clinical and follow-up data were collected from a review of the patients' records and by contacting the referring physicians. Follow-up data collected included the clinical management of OGIB, episodes of rebleeding after CE, and the length of the follow-up period. Rebleeding was defined as the visible passage of blood or positive fecal occult blood testing with a drop in hemoglobin after the CE examination.

### Statistical analysis

The results were presented as the mean ± standard deviation for quantitative data or a frequency (percentage) for categorical data. Data were analyzed using a chi-square test a Yates chi square test or a Fisher's exact test as appropriate. A *p *value of less than 0.05 was considered statistically significant.

### Ethical consideration

This study was carried out in accordance with the Declaration of Helsinki (1989). Ethical permission was granted by the Kanto Medical Center NTT EC, Tokyo, Japan, Ethics Committee. Written informed consent was obtained from all patients for their involvement in this study.

## Results

All the CE examinations were performed without any complications. None of the patients developed intestinal obstruction during the examination and none required endoscopic or surgical removal of the capsule. A total of 77 patients with OGIB whose follow-up data were available had undergone CE, and their demographic data are shown in Table 1. In 52 of the 77 (67.5%) patients, the capsule passed into the cecum within the recording time; in the remaining 25 (32.5%) patients, the capsule was spontaneously eliminated within 2 weeks after the CE. The mean follow-up period was 11.6 months (range: 5–34 months) after the CE examination. The mode of presentation was overt bleeding in 60 (77.9%) patients and occult bleeding in 17 (22.1%) patients. The mean lowest hemoglobin concentration was 7.7 ± 2.9 g/dl (range: 3.5–17.5 g/dl). Forty-one (53.2%) patients required a transfusion of packed red blood cells for the treatment of anemia as a result of GI bleeding.

**Table 1:** Patient characteristics

	n (%)
No. of patients	77
Gender (M/F)	54 (70.1)/23 (29.9)
Mean age, years ± SD	58.4 ± 18.4
Exam complete to colon	52 (67.5)
Bleeding (overt/occult)	60 (77.9)/17 (22.1)
Mean hemoglobin concentration, g/dl ± SD	7.7 ± 2.9
Mean follow-up period, months	11.6

The details of the CE finding in the 77 patients are listed in Table 2. CE identified clinically significant findings that were thought to be the sources of OGIB in 45 (58.4%) patients. On the other hand, CE showed insignificant findings in 32 (41.6%) patients. Small bowel ulcers were the most common finding. The second most common finding was small bowel angiodysplasia.

**Table 2:** Findings of capsule endoscopy and rebleeding rate

Finding	n	Finding	n
Significant		Insignificant	
Overall	45 (58.4%)	Overall	32 (41.6%)
Ulcer	16 (35.6%)	Erosion	16 (50.0%)
Angiodysplasia	13 (28.9%)	Small ulcer	7 (21.9%)
Tumor	8 (17.8%)	Red spot	3 (9.4%)
Bleeding without identifiable lesion	7 (15.6%)	SMT/polyp	2 (6.3%)
Crohn's disease	1 (2.2%)	Negative	4 (12.5%)

Note: SMT, submucosal tumor

Twenty-eight (36.4%) patients experienced rebleeding during the follow-up period. Most of the rebleeding occurred within the first 6 months after CE, and the mean interval between the rebleeding and the CE examination was 5.7 months. During the follow-up period, 12 of the 45 (26.7 %) patients with significant findings developed clinical rebleeding. On the other hand, 16 of the 32 (50.0 %) patients with insignificant findings developed clinical rebleeding. The probability of rebleeding was significantly higher among the patients with insignificant findings than among those with significant findings (*p *= 0.036).

**Figure 1:**
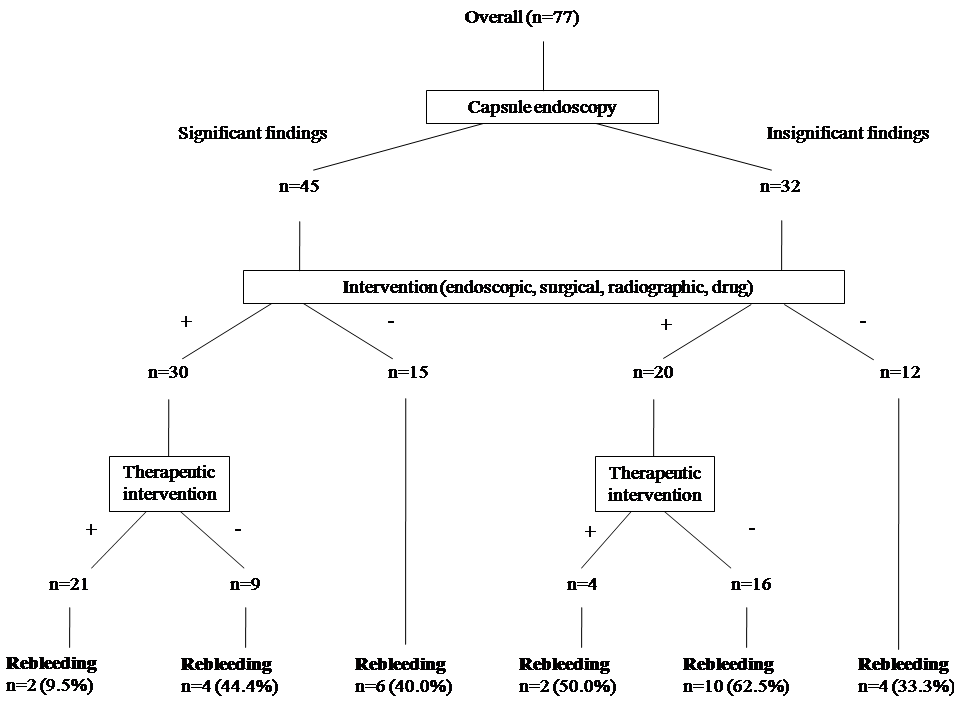
Comparison of the rebleeding rates for each capsule finding with or without intervention.

In total, 50 of the 77 (64.9 %) patients underwent further examinations and interventions after the CE; these examinations included endoscopic, surgical, radiographic, and drug therapy. Of the 50 patients who underwent further examinations and interventions, 25 patients underwent therapeutic interventions. Therapeutic interventions included endoscopic cautery, surgical resection, angiographic embolization, and specific drug therapy, such as the use of mesalazine for the treatment of Crohn's disease. In this study, we defined nonsteroidal anti-inflammatory drugs (NSAIDs) withdrawal for NSAIDs-induced enteropathy as a specific drug therapy. Thirty of the 45 (66.7 %) patients with significant findings underwent further interventions; of these patients, 21 (70.0 %) underwent therapeutic interventions. Among the patients with significant CE findings, the rebleeding rate of the patients with therapeutic intervention was significantly lower than that of those without intervention (9.5% vs 40.0%, *p *= 0.046). On the other hand, 20 of the 32 (62.5 %) patients with insignificant findings underwent further interventions and 4 of these 20 (20.0%) patients underwent therapeutic interventions. The rebleeding rate between the patients who did and did not undergo intervention was not significantly different. The results are presented in Figure 1. Twenty-seven patients did not undergo further intervention after CE examination. The main reasons for not undergoing further examinations and interventions were the CE findings were too small to justify further interventions (n = 10), the absence of clinical rebleeding for a notable period (n = 10), co-morbidity (n = 4), and patients' refusal (n = 3).

The capsule findings, the subsequent procedures, and the rebleeding rate after further intervention are summarized in Table 3. The highest rate of rebleeding was seen in patients with ulcers (31.2%) and angiodysplasia (30.8%). Of the patients who were found to have an ulcer, the rebleeding rate of the patients who did not undergo further intervention was higher than that of those who did undergo further intervention (*p *= 0.100). The patients with angiodysplasia tended to develop clinical rebleeding (50.0 %) despite endoscopic intervention, including cautery. As expected, none of the patients who were found to have a tumor and who underwent surgical intervention subsequently developed rebleeding. Of the 7 patients with bleeding without any identifiable lesions, 5 patients underwent further procedures. Further examinations resulted in diagnoses of small bowel angiodysplasia (n = 1), NSAIDs-related enteropathy (n = 1), hemosuccus pancreaticus (n = 1), colonic diverticula (n = 1), and unknown (n = 1). Among them, rebleeding was observed in 2 patients diagnosed with small bowel angiodysplasia (endoscopic cautery was performed) and colonic diverticula (an additional colonoscopy was performed), respectively.

**Table 3:** Rebleeding rate on follow-up procedures for significant capsule finding

Finding	Endoscopic	Surgical	Radiographic	Drug	Without intervention	Total
Overall	5/12 (41.7%)	0/9 (0%)	0/1 (0%)	1/8 (12.5%)	6/15 (40.0%)	12/45 (26.7%)
Ulcer	0/4 (0%)	0	0	1/5 (20.0%)	4/7 (57.1%)	5/16 (31.2%)
Angiodysplasia	2/4 (50.0%)	0/2 (0%)	0	0/1 (0%)	2/6 (33.3%)	4/13 (30.8%)
Tumor	1/1 (100%)	0/7 (0%)	0	0	0	1/8 (12.5%)
Bleeding without identifiable lesion	2/3 (66.7%)	0	0/1 (0%)	0/1 (0%)	0/2 (0%)	2/7 (28.6%)
Crohn's disease	0	0	0	0/1 (0%)	0	0/1 (0%)

Of the rebleeding cases, 4 patients in whom the source of the rebleeding was detected during further examinations had no small intestinal lesions. The sources of bleeding in these patients were colonic diverticula (n = 2), gastric angiodysplasia (n = 1), and hemosuccus pancreaticus (n = 1).

## Discussion

The precise role of CE in the diagnostic algorithm of OGIB has yet to be determined. Moreover, despite the higher diagnostic yield of CE compared with other diagnostic tools, the actual impacts of this examination on patient outcome remain poorly defined. As CE itself is a purely diagnostic modality, the improvements in bleeding parameters cannot be directly attributed to CE. Instead, it is likely that CE directs clinicians to the most appropriate definitive therapy. If this therapy is effective, the rebleeding rate of the patients ought to improve. We determined the clinical outcome of patients with OGIB after CE using the rebleeding rate as the primary outcome. Among the patients with significant CE findings in this study, the rebleeding rate of the patients who underwent therapeutic intervention was significantly lower than that of those who did not undergo intervention (9.5% vs 40.0%, *p *= 0.046). Thus, a clinically significant improvement was seen among the patients with significant CE findings who underwent therapeutic intervention. In previous reports, the presence of significant CE findings was correlated with rebleeding [15,16]. However, we proved that the rebleeding rate of patients with significant CE findings was considerably reduced with appropriate interventions after CE.

On the other hand, patients with insignificant CE findings had a high rate of rebleeding during the follow-up period. To say that the CE examination is not useful for these patients with rebleeding was not overstating the case. There are two main reasons why CE may be unable to identify significant lesions. The first reason is that the CE may miss a small intestinal lesion that accounts for the bleeding. In our study, jejunal diverticula and a GI stromal tumor were not visible on the CE images. Previous studies have also reported that tumorous lesions were missed during CE [17,18]. Moreover, we experienced two cases in whom small intestinal ulcers were detected in the distal part of the ileum using double-balloon enteroscopy. The CE examinations had missed these lesions because the capsules had not reached the cecum. Thus, the CE findings were negative in these cases. In cases where the CE results are negative but a high clinical suspicion of a small bowel lesion remains, further examinations should be considered. The second reason is that the source of the bleeding may not exist in the small bowel from outset. In this study, four patients with rebleeding, in whom the source of the bleeding was detected during further examinations did not have any small intestinal lesions. The sources of bleeding in these patients were colonic diverticula (n = 2), gastric angiodysplasia (n = 1), and hemosuccus pancreaticus (n = 1). Thus, the source of OGIB does not always originate in the small bowel.

The diagnostic yield of CE in patients with OGIB has been described in previous reports [8,10,19-22]. The range of the yields varies widely (31–92%) because of differences in the definition of positive findings and the patient characteristics. In the present study examining 77 patients, the overall diagnostic yield was 58.4% for the significant findings. Ulcers were the most common finding. This result differed from that of many previous studies, in which angiodysplasia was the most common finding [19,22,23]. This discrepancy may be due to ethnical differences. Several studies in Japanese populations have shown ulcers to be the most common finding among small intestinal lesions [24,25]. Ulcerations induced by the use of NSAIDs typically resolve upon withdrawal of the medications in most cases. In this study, only 1 of 4 patients developed rebleeding. However, specific therapies have not been established for nonspecific ulcers. Therefore, among these cases, the rebleeding rate may be relatively high even though the source of bleeding has been identified.

In this study, we evaluated the clinical impact of CE for directing interventional therapy in patients with OGIB and reported the long term results of this strategy using the rebleeding rate as the primary outcome. This important issue has been rarely studied to date; however, some previous reports have described similar results. Recently, Garcia-Compean et al. reported that a significantly reduced rate of recurrent bleeding (6%) was observed in patients with positive CE findings who underwent specific treatment in a study of 40 patients with OGIB [26]. Delvaux et al. also reported similar results: among 18 patients who were treated for intestinal lesions that were detected by CE, only one patient (5%) relapsed during a 1 year follow-up period [27]. Although the details of their treatments were not exactly the same as ours, their results were very similar. Moreover, when lesions observed at CE were divided into highly relevant (P2) or less relevant (P0, P1) according to the possibilities of their being responsible for the GI bleeding, highly relevant lesions were more frequently classified in true-positive cases and led more frequently to therapeutic decisions, compared with less relevant lesions [28]. The named P2 lesions proposed by some authors correspond to significant findings of the present study. These results suggest that this classification of CE findings may assist clinicians in making appropriate therapeutic decisions and may improve the clinical outcome of patients with OGIB.

The selection of patients who underwent subsequent interventions must be considered. Patients who had significant co-morbidity and were on aspirin or anticoagulants might have a high risk of rebleeding. In a recent report, Sidhu et al. demonstrated that co-morbidity was a significant predictive factor of a positive diagnostic yield [26], and this result might be related to the rebleeding rate in this patient population. However, in this study, only 1 of the 4 patients who had co-morbidities developed rebleeding. Moreover, none of the patients did not undergo therapeutic intervention because of co-morbidity or the use of aspirin/anticoagulants. Therefore, co-morbidity and the use of aspirin/anticoagulants did not affect the decision to undergo intervention and were not associated with a high risk of rebleeding in this study. Further CE studies are required to confirm these results.

This study has several limitations. First, this study was not a randomized trial. Second, our results may be biased because we tended to follow up our own patients more accurately whereas the information from other hospitals was often incomplete, probably because the referring physicians tended to inform us about their positive clinical results only. Unfortunately, we could not evaluate the hemoglobin values after intervention in this study. Third, our study lacked objective criteria for performing the follow-up examinations. A prospective study with objective criteria is needed to determine the true efficacy of pursuing further interventions after CE.

While swallowing disorders and pacemaker implantation were regarded as exclusion criteria in the present study, these factors are no longer regarded as contraindications for the performance of CE. Actually, none of the patients considered for enrollment in the present series had suspected swallowing disorders or implanted pacemakers.

## Conclusion

We evaluated the follow-up results of patients with OGIB to determine which management strategies after CE reduced rebleeding. The rebleeding rate was significantly higher among patients with insignificant CE findings than among those with significant CE findings. Among the patients with significant CE findings, the rebleeding rate of the patients who underwent therapeutic intervention was significantly lower than that of those who did not undergo intervention. Follow-up and further aggressive interventions are necessary for patients with OGIB whose CE findings are significant to reduce the chance of rebleeding. Moreover, patients with insignificant CE findings should undergo careful follow-up, keeping in mind that the bleeding may not originate from within the small bowel.

## Competing interests

The authors declare that they have no competing interests.

## Authors' contributions

HE analyzed the capsule endoscopies, collected the clinical data and wrote the manuscript, with contributions from NM and AN. AN was responsible for the design of the study and collected the clinical data. MI, KF and MY performed the statistical analyses. KA, TO, TY, MA, KH and TT analyzed the capsule endoscopies and participated in the design and coordination of the study. All authors read and approved the final manuscript.

## Pre-publication history

The pre-publication history for this paper can be accessed here:


